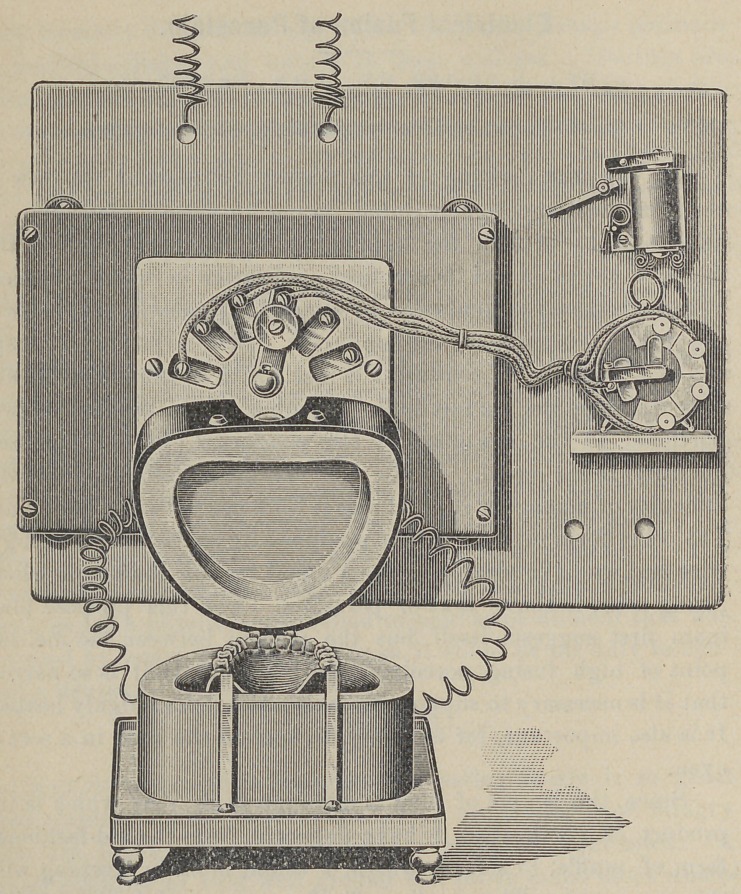# Electrical Fusion of Porcelain

**Published:** 1895-02

**Authors:** L. E. Custer

**Affiliations:** Dayton, O.


### Electrical Fusion of Porcelain.

BY L. E. CUSTER, B.S., D.D.S., DAYTON, O.

Read before the Ohio State Dental Society, December, 1894.

At the last meeting of this society I explained the law of electrical heat and demonstrated its use for fusing platinum. I have now to explain the application of electrical heat to the fusing of porcelain and its practical application in dental practice. When electricity is conducted by a metal it produces heat according to the resistance of the conductor. When the current leaps across a break in the conductor it meets with so great a resistance as to develope the highest heat. In other words, the heat is proportional to the resistance of the conductor, the voltage and quantity being equal. While the arc gives sufficient heat the difficulty of managing it precludes it from use in fusing porcelain. The other form of heat is that produced by electrically heating a wire and we have simply to use a metal for the conductor whose fusing point is above that of porcelain. For this purpose platinum first suggests itself, but the margin between the melting point of high fusing porcelain and platinum itself is so narrow that it is necessary to support the wire while it is so highly heated. It is also important, for economy, to enclose the heat in a sort of oven.

Since the source of heat is different from that which is the product of combustion I have departed from the old-fashioned form of muffle, to a form which, I think, more in keeping with this new agent. The small muffle for crown and bridge-work is made essentially of a plain base upon which rests a removable cup-shaped cover. In the upper part of the cover is a small opening for observing the fusing process. The advantage of this form is that the most delicate crown and band can be placed in position upon the base for fusing without disturbing their arangement. In most muffles it is necessary to slide the piece in on a tray, during which there is danger of jarring the parts.

The cover being hinged it is easily guided to its proper position where the electrical connection with the base is automatically made.

The amount of current for operating the small oven is equal to about a hundred candle lamp. While the 110-volt current furnishes an abundant current for the small oven so that the current passes through the cover and base as a single current, this arrangement will not give sufficient heat for a large muffle. It requires so large an amount of wire that the resistance is too great. This electrical problem governs the form of the oven for a full case. The large oven is constructed very much in the form of an ordinary vulcanite flask, the upper section being a duplicate of the lower. In the upper section of this also is a small opening for observation. By the new mode of wiring the large oven another advantage is gained, the upper section may be removed and its current broken without cutting off the current from the lower. So that a case can only be dried out as if it were in an open tray, but the heat raised to that point where flaking occurs, so that this can be repaired and the baking proceeded with without any interruption. It might be noticed incidentally that the arrangement furnishes as well a most perfect appliance for heating up and soldering cases of all kinds.

The operation of the electric oven is quite simple. It is furnished with a rheostat so that the heat may be as gradually and accurately raised as a vulcanizer; not only so but the heat can be cut off instantly.

If it is desired to raise the heat without the hand lever it may be done absolutely by clock work. Let one wire of the rheostat connect with a lever attached to the minute hand stem of a clock and place contact plates at such intervals on an insulated dial plate as you may wish the current increased. The last plate is to be placed at that point where it is desired to turn the current off. I find that in spite of varying currents this gives results that are quite accurate.

One inexperienced in porcelain work will find difficulty in telling the exact fusing heat. To meet this I have also devised an automatic cut-off. A plug of fire-clay which fits the hole for observation has running through it two platinum terminals which are connected with a magnet operating a cut-off. A button of the same body, or gum as that used in the case, is laid upon the lower terminal of the plug. When the fusing point is reached and the button melts the upper terminal, by its weight, is allowed to come in contact with the lower whereupon the circuit is closed and the magnet releases the contact spring at which the current is cut off from the whole instrument.

The time regulator and thermostatic cut-off are not essential to the oven, however, in fact it is such a pleasure to operate it that the best practitioners, I think, will prefer the more certain method of operating it by hand. When an operator has an instrument which instead of confining himself to a hot room and all the disagreeable things connected with it known to you all, when instead he can place upon his operating table and with his finger upon the button and at will produce a clean heat up to the melting point of platinum, a disagreeable task becomes a fascinating pleasure. I have time and again fused a case at the same time I was making a gold filling. While a case may be fused with this appliance in from five to ten minutes starting from a cold muffle, for reasons known to every dentist, it is better to raise the heat gradually. In the time attachment I have fixed the time of fusing at thirty minutes, but this may be changed at pleasure.

Since the case is enclosed and the light is the same from all directions, the glaze cannot be detected as in an old fashioned muffle. But there is another and, I think, a more accurate method of telling the fusing point. The eye being but a few inches from the piece, it is able to accurately observe the different stages of the fusing. He can see the different molecules as they coalesce. During the first stage the body will appear like snow. As the heat is increased it undergoes contraction, during which time fissures form. The difference in color between the body and the teeth is still well marked. It is due to the loose texture of the unfused body, but after a few moments the white and granular appearance of the body begins to deaden and becomes like the teeth. The particles are coalescing. If the current be now turned off it will be to a "biscuit," but by continuing the heat a little longer, till nothing but the outlines of the teeth are distinguishable, the case is fused. The current should be immediately cut off and the stopper inserted. If you have only brought to a "biscuit" the stopper may be left out when the case may be removed in thirty to forty minutes, but if it is for a full fuse the stopper should be inserted and the case allowed to become perfectly cool, which requires about two hours. If it is desired to cool less rapidly, a glass globe placed over the oven will prolong the time to three hours and a half.

The advantages of the electric oven for porcelain work may be summed up as follows:

The heat is high enough to fuse any porcelain used in dentistry.

The heat being derived from an electrically heated platinum wire, itself a noble metal, invested in an infusible material, is perfectly free from any gas so common with most furnaces.

The ease with which the heat can be controlled with a rheostat.

The perfection with which the heat can be cut off, so that there is no danger of over-heating.

Instead of removing the case from the heat, the heat is removed from the case, which prevents a change of position of the teeth as well as checking.

The freedom from noise, dirt and heat of the room as well as the comparatively small cost of operating it.

DISCUSSION.

Dr. Grant Molyneaux: I see I am down to discuss this paper, I can't see that I can discuss it very much, but the method seems to me to be the way we long have sought, something that can be thoroughly controlled and easy of manipulation. It don't seem to me that it makes any difference in the case as to the character of the heat or that the different kinds of heat would make any special difference. The heat from a coke furnace would probably not be different from the heat of this electric furnace up to the point of incandescence. The coke furnace or gas furnace requires an undivided attention during the entire process of firing. The danger that arises out of what is called gasing, is the discoloration that occurs and gives the continuous gum its dead appearance that is done so frequently with a gas furnace. Our gas generated must necessarily come from the gas generated during the combustion of the gas or coke. This discoloration must be produced before the piece reaches the point of incandescence, and I think in this furnace it would require almost as much time to burn a piece as it would in the coke furnace, that is, after raising the heat to a certain point. In the ordinary coke furnace we must start from the outside of the muffle and heat the whole inside of the furnace, everything must be incandescent. In making continuous gum work we have our piece ready and set it near the heat to dry out gradually, and as the muffles begin to redden we introduce the work. The dangers are from introducing it into the furnace before it is hot.

The advantage of this furnace is, there is no possibility of gas. The heat is so far under control it requires no attention from the dentist. It can get gradually warm, that is, kept up a sufficient time to dry every bit of moisture out of the body and after that moisture is out the heat can can be raised as rapidly as possible until the point of fusion is reached.

The succes of continuous gum work lies in two points : first, in taking it out at the proper temperature to maintain an adaptation of the platinum, and the compensation for the effect the shrinkage would have on the palate, in hard points upon which there was any shrinkage the adaptation must be altered a little. This can be done without injury to the plate. Aside from this trouble and the trouble of repairing, that is, with the old furnace and the labor connected with making the fire, continuous gum work is a more certain work than any other we have. It is certainly the highest type of mechanical denture. It is more cleanly, and it is durable. The tissues retain their health and the ridge retains its form under a platinum denture longer than any other plate.

With the furnace Dr. Custer has introduced to this society, repairing would be a matter of little consideration. If a plate happened to fall and something broke off it would be easily restored. From the fact that there is no gas about it, there is no danger of free gas attacking the body. There need be no apprehension of that. I think the introduction of this into the laboratory of the dentist will revolutionize that department. It decreases the trouble arising from accidents—if you break a tooth it does not have to go through the big coke furnace when you haven't time for it, and don't get paid for it, for you can do it without loss of time. I think it is one of the grandest achievements that has been made in the profession recently, and I think Dr. Custer deserves a great deal of credit for having solved this problem.

Aside from the continuous gum work there are many features that might be spoken of as a recommendation of the furnace; in considering bridge-work and crown, in gold plate, platinum plate and porcelain, which we can do without loss of time.

I have had a good deal of experience with the different kinds of furnaces used. I have not had experience with the recent furnace of Dr. Land's, but the coke furnace in nearly every form, and and the gas furnace I have had experience with and I have never seen the same color or strength obtained through gas furnaces or from patent coke furnaces. The color is different. I don't believe there is any difference in the heat up to the point of incandescence.

Porcelain work depends for its strength on the perfect union of every molecule of that body, and the perfect union of that body to the platinum plate.

These pieces are glazed throughout and it requires a considerable amount of strength to break them. Those made by the old process are not solid throughout. The heat seems to be the best heat you could possibly have, because it is under control. We can raise the temperature gradually at will. You can shut off the current and the heat stops and it cools down. I expect great things from this furnace.

Dr. Frank Hunter, Cincinnati: I have been in the profession for a few years and I consider myself quite a young man, in fact, quite a boy. I have had, in former years, considerable experience in this, and up to the time I practically stopped mechanical dentistry I had my share of experience in it, but seeing what I have seen here and hearing what I have heard, I have come to the conclusion I must be a back number. Dr. Custer is one of those electrical cranks that there is no telling what he is going to do next. If he keeps on at this thing I don't know where he is going to land, but it is perfectly rational. It is certainly the greatest advance I have ever seen.

Dr. Ames: Dr. Custer was at my office a couple of months ago and told me what he had, and I would not let him leave town until he came to my office and hitched it up to the electric light and I rubbed his fur down then and there. It is a greater step in advance than anything that has been brought out in a long time. It accomplishes more at one sweep you might say. It applies not only to continuous gum work but to crown-work and bridge-work. We can do more artistic work than we could before. There is no question but this is better than the porcelain and gold work we have been putting into our mouths, and I think great credit is due Dr. Custer for what he has done in this work, and I can say nothing except to commend Dr. Custer's work. The positive freedom from gas is a very great point. All I can say is, I would take off my hat to Dr. Custer.

Dr. C. H. Harroun, Toledo: I have been in the business since 1858, when I purchased an old muffle furnace from Cincinnati. I have gone through all the processes, spoiling things. I think now the time has come when I can begin again as a boy. I think any man who has made a piece of continuous gum work will say that a good thing has been accomplished. There may be something arrived at that will make it more sure of success, but it seems to me we have come to the point where perfection is very near.

I had the pleasure a number of years ago, as one of the board of examiners of this state, to inquire into the qualifications of a gentleman that came down from Westerville up here. He had a family of children going to school and worked hard trying to support them and give them an education. His name was Custer. I voted to give him a certificate to practice dentistry. That is a little history of the Custer family. If I am not mistaken this Dr. Custer is one of the boys that was going to school them. His father was a workman in those days—rough and uncouth when he first went to work. He was giving his family an education so they could be something in life, and I am very much pleased with the results coming from that work, and I feel proud that we have got a man in our State capable of bringing out such an appliance. Ohio has taken the lead in many things, and we have taken the lead in this thing to-day.

Dr. H. A. Smith, Cincinnati: I suppose stories about the Custer family are in order. I was very much amused a few days ago by the estimate of one Custer about the other. I

asked, "how is Levitt getting on?" He says, "I don't know that there is much good in him, he has gone daft on electricity. I am afraid he is going to be a failure."

I could tell another story about the Custer family. You know Professor Wright. He tells the story that all our students take their notes in Greek. He boasted of this. One day in his lecture in physiology he left his text-book at home and asked Custer, a brother of this Dr. Custer, for his text-book, and he had taken all his notes in Greek.

Dr. Geo. L. Field, Detroit: I am glad I came down here. I have done a great deal of porcelain work. I have a furnace that weights 500 pounds, and I have worked from the time I started out at night until broad day light in the morning. I first began with Dr. Spaulding in 1861, when a patent was obtained for making continuous gum, and Dr. Spaulding wrote Dr. Allen telling him to send the furnace and if he liked it he would buy the patent. I was one of the boys that helped make fires in the furnace. The first work was done in St. Louis in 1862. Since then I have done a great deal of the work and have taken a great deal of interest in it. The labor of making continuous gum work has probably prevented many men from doing it who would otherwise do it. It required a good deal of labor and time and money to do this thing, and they thought it wouldn't pay them, especially when living in a community where people couldn't afford to pay for their work so much money. I saw this running down from a large furnace weighing 400 pounds gradually getting down to a small furnace until we got the Ambler Tees furnace that we thought a good thing. From that it has gone down to a still smaller thing in the way of a little furnace that works admirably, but when I see this little toy furnace I haven't a word to say. This is beautiful. You won't see one case in five hundred that will come it out that way without any cracks in it. You can't do that with the old furnaces we use to-day. There was always more or less danger of over-heating, but you don't get as good work as if you bring it to what we call a biscuit heat; so that the bodies will show granules as the dew on the grass in the morning. That seems to be done very beautifully so that this gum can be beautifully put on. I am very glad I came to Ohio.

Dr. Taft: I can conceive a great many purposes for which that can be used in addition to porcelain work. I don't see why it wouldn't be a good furnace for melting gold and making alloys and many kinds of work. The difficulty many times in melting and alloying gold is the foreign substances in the fire that get in the gold and which interfere with and mar the results. Nothing of that kind could occur here.

Dr. Otto Arnold, Columbus: I don't know anything that could be said just now that would enhance the value of this valuable invention.

Dr. Arnold moved that a committee of three be appointed to procure a suitable medal to be presented to Dr. Custer as a testimonial from the society, of appreciation for the valuable appliances invented by him. The President appointed Drs. O. Arnold, J. Taft and C. R. Butler on this committee.

## Figures and Tables

**Figure f1:**